# Pericardiectomy using an ultrasonic scalpel in patients with constrictive pericarditis

**DOI:** 10.1093/icvts/ivaf025

**Published:** 2025-02-08

**Authors:** Takashi Yoshinaga, Jun Takaki, Kosuke Nakata, Takafumi Hirota, Hideaki Hidaka, Tatsuya Horibe, Ken Okamoto, Toshihiro Fukui

**Affiliations:** Department of Cardiovascular Surgery, Kumamoto University Hospital, Kumamoto, Japan; Department of Cardiovascular Surgery, Kumamoto University Hospital, Kumamoto, Japan; Department of Cardiovascular Surgery, Kumamoto University Hospital, Kumamoto, Japan; Department of Cardiovascular Surgery, Kumamoto University Hospital, Kumamoto, Japan; Department of Cardiovascular Surgery, Kumamoto University Hospital, Kumamoto, Japan; Department of Cardiovascular Surgery, Kumamoto University Hospital, Kumamoto, Japan; Department of Cardiovascular Surgery, Kumamoto University Hospital, Kumamoto, Japan; Department of Cardiovascular Surgery, Kumamoto University Hospital, Kumamoto, Japan

**Keywords:** constrictive pericarditis, pericardiectomy, ultrasonic scalpel

## Abstract

**OBJECTIVES:**

Pericardiectomy using an ultrasonic scalpel is the effective operative treatment of chronic pericarditis. However, no consecutive case series of radical pericardiectomy using an ultrasonic scalpel has been reported.

**METHODS:**

We retrospectively reviewed the records of 10 patients who underwent a radical pericardiectomy with an ultrasonic scalpel between April 2015 and March 2024. Preoperative and postoperative variables were reviewed.

**RESULTS:**

The median age was 65 years, and 3 patients were female. Three patients had preoperative atrial fibrillation. Forty percent of patients were in NYHA class 3–4. Three patients underwent urgent operations. An ultrasonic scalpel was used for the dissection and resection of thickened parietal pericardium and visceral pericardium around the heart except in the area of the posterior left atrium in all patients. Three patients underwent the off-pump technique. Coronary artery bypass grafting and tricuspid valve annuloplasty were performed concomitantly in 3 and 1 patients, respectively. There were no operative deaths. Mediastinitis and acute kidney injury were observed in 1 patient each. One patient died of a stroke during the follow-up period. The other patients were in NYHA class I.

**CONCLUSIONS:**

Radical pericardiectomy is essential in patients with constrictive pericarditis. An ultrasonic scalpel is a useful tool with which to perform a radical resection of pericardium.

## Graphical Abstract

**Figure:**
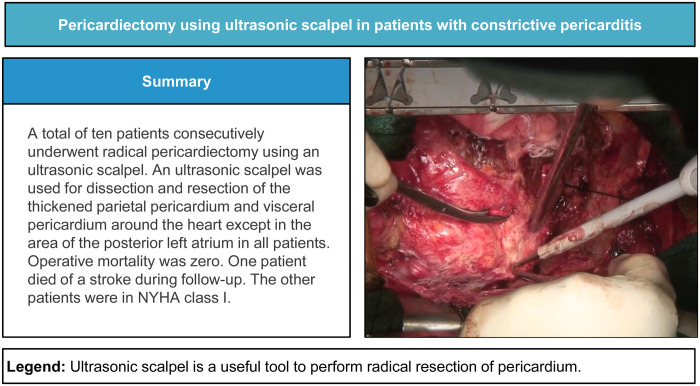

## INTRODUCTION

Constrictive pericarditis is a chronic and progressive inflammatory disease of the pericardium that involves pericardial scarring and fibrous thickening of the pericardium [1]. Although rare, constrictive pericarditis is one of the long-standing causes of heart failure. Although most patients with pericarditis are managed medically with diuretics, pericardiectomy is the definitive treatment for patients with uncontrolled heart failure. However, the early postoperative mortality of pericardiectomy is high, ranging from 2% to 15% [2]. Adequate pericardial resection is critical to prevent persistent haemodynamic compromise and symptoms due to residual constrictive physiology [3]. Failure to free the inferior aspect of the ventricles and remove the diaphragmatic pericardium may result in residual constriction and recurrent symptoms [4].

There have been only a few case reports on the feasibility of using an ultrasonic scalpel to dissect thickened pericardium in patients with constrictive pericarditis [5, 6]. Uchida *et al.* [5] reported that using an ultrasonic scalpel made it relatively easy to find the correct plane to avoid injuring the coronary arteries even though the pericardium was strongly adherent to the myocardium. We have used an ultrasonic scalpel for the treatment of constrictive pericarditis to achieve complete resection of thickened pericardium. In this study, we evaluate the effectiveness of using an ultrasonic scalpel for constrictive pericarditis.

## PATIENTS AND METHODS

We studied 10 consecutive patients who underwent ultrasonic scalpel pericardiectomy for the treatment of constrictive pericarditis at the Kumamoto University Hospital. Patients who underwent concomitant procedures were included in this study. The study was approved by the research ethics committee at Kumamoto University (Approval No. 3070). The institutional review board approved this retrospective study and waived the requirement for written consent.

Our strategy for pericardiectomy was to achieve complete resection of the thickened pericardium with or without cardiopulmonary bypass. Cardiopulmonary bypass was necessary for cardiac decompression and circulatory support, especially in those patients whose lateral and infero-posterior pericardium was extremely thickened and calcified. Cardiopulmonary bypass was also required for concomitant procedures such as valve replacement.

A median sternotomy was performed in all patients. Our operative strategy and methods are similar to those described by Hemmati *et al.* [3]. The pericardial incision was started in the midline (Video 1). First, an electric cautery was used to create a dissection between the pericardium and epicardium. Once the correct dissection plane could be observed, dissection was performed with an ultrasonic scalpel. Our intention was to dissect the plane between the pleura and the pericardium; however, the pleural cavity is usually opened on both sides because the adhesion between the pleura and the pericardium is tight. Resection of the anterior and lateral sides of the pericardium was performed until the phrenic nerves were seen. The diaphragmatic pericardium was dissected from the inferior surface of the left and right ventricles using a heart positioner normally used for off-pump coronary artery bypass grafting. The diaphragmatic pericardium was resected from the underlying muscular and fibrous portions of the diaphragm. We resected the diaphragmatic pericardium until the inferior pulmonary veins could be seen. The right and left sides of the diaphragmatic pericardium were also resected until the phrenic nerves were visible. The fibrous tissues around the superior and inferior vena cava were also resected.

Next, we removed as much as possible of the thickened epicardium that was affected by the inflammation of the pericardium. An ultrasonic scalpel is particularly useful for epicardial resection because it causes less bleeding and does not induce ventricular arrythmias. We do not perform the “waffle procedure [7]” unless the epicardial adhesion is dense and the epicardium is difficult to remove completely. Concomitant procedures were performed after completion of the pericardiectomy.

All patients underwent echocardiography 2–3 days before the operation and 7–12 months after the operation [median 12 months (10.5–12.0)].

All statistical analyses were performed with the StatView 5.0 software package (SAS Institute Inc., Cary, NC, USA). Continuous variables are presented as the median and interquartile range or the mean ± standard deviation. Continuous variables were compared using paired *t*-tests. Differences were considered significant at *P* < 0.05.

## RESULTS

We performed ultrasonic scalpel pericardiectomy in 10 patients between April 2015 and March 2024. Pericardial resection was completed in all cases. The preoperative characteristics of the patients are listed in Table 1. None of the patients had had previous cardiac operations. Three patients urgently underwent operations because they had acute heart failure with catecholamines.

**Table 1:** Preoperative characteristics of the patients

Variable	All patients (*n* = 10)
Age (years)	65 [55.8–68.5]
Women	3 (30%)
Body mass index	21.9 ± 6.4
Hypertension	4 (40%)
Diabetes mellitus	5 (50%)
Hyperlipidemia	2 (20%)
Smoking history	7 (70%)
Previous stroke	1 (10%)
Peripheral vascular disease	0
Haemodialysis	2 (20%)
Prior myocardial infarction	0
NYHA class 3–4	4 (40%)
Atrial fibrillation	4 (40%)
Previous cardiac surgery	0

Data are presented as median [interquartile range] or as number (percentage).

NYHA: New York Heart Association

Operative and postoperative data are shown in Table 2. Concomitant procedures were performed in 4 patients, including coronary artery bypass grafting in 4 patients, aortic valve replacement in 1 patient, and tricuspid valve repair in 1 patient. Cardiopulmonary bypass was used in 7 patients. Of those, 3 patients did not undergo any procedures other than pericardiectomy. There were no operative deaths. In addition, no patients experienced major postoperative complications such as stroke, bleeding requiring re-exploration, low output syndrome, perioperative myocardial infarction or respiratory failure. However, 1 patient required haemodialysis and 1 patient had mediastinitis.

**Table 2:** Operative and postoperative data

Variable	All patients (*n* = 10)
Concomitant procedures	4 (40%)
Without cardiopulmonary bypass	3 (30%)
Operation time (min)	248 (211.5–391.8)
Cardiopulmonary bypass time (min)	117 (107.5–137)
Operative deaths	0
Stroke	0
Use of haemodynamic support device	0
Respiratory failure	0
New haemodialysis	1 (10%)
Mediastinitis	1 (10%)
New atrial fibrillation	1 (10%)
Bleeding requiring re-exploration	0
Postoperative ICU stay (day)	3 [2–5]

Data are presented as median [interquartile range] or as number (percentage).

ICU: intensive care unit.

Pre- and postoperative echocardiographic data are shown in Table 3. Both diastolic and systolic dimensions of the left ventricle were significantly improved. Similarly, both end-diastolic and end-systolic left ventricular volumes were significantly improved. However, the left ventricular ejection fraction did not change. Most of the parameters of left ventricular diastolic function were significantly improved after the operation.

**Table 3:** Pre- and postoperative echocardiographic data

	Preoperative	Follow-up	*P*-value
Left ventricular diastolic dimension (mm)	34.6 ± 7.3	41.6 ± 5.0	0.010
Left ventricular systolic dimension (mm)	22.9 ± 5.2	27.5 ± 3.6	0.013
Left ventricular end-diastolic volume (ml)	45.2 ± 17.2	65.2 ± 17.5	0.012
Left ventricular end-systolic volume (ml)	18.9 ± 7.6	25.9 ± 7.6	0.018
Ejection fraction (%)	58.2 ± 4.7	60.4 ± 6.1	0.714
E/A ratio	2.4 ± 1.6	1.2 ± 0.4	0.022
Deceleration time (ms)	132.7 ± 41.5	195.6 ± 67.1	0.028
*E*/*e*′ ratio	8.5 ± 3.3	9.0 ± 3.2	0.566

Values are means ± SD.

## DISCUSSION

Surgical resection represents a fundamental approach to the treatment of constrictive pericarditis. The primary objective of pericardiectomy is to achieve complete resection. The majority of surgeons perform a pericardial resection that extends to the phrenic nerve on both sides. Nevertheless, this step may lead to the persistence of left ventricular dysfunction. To achieve complete resection, it is essential to remove the pericardium from the diaphragm to the posterior aspect of the heart. We use cardiopulmonary bypass in patients whose haemodynamics are unstable during the resection of the posterior side of the heart. Furthermore, it is crucial to remove as much of the epicardium as possible. The procedure carries a significant risk of bleeding from the ventricular muscle and damage to the coronary arteries. In our institute, an ultrasonic scalpel has been used to resect the pericardium and epicardium. Uchida *et al.* [5] reported its usefulness in a case report and described that haemostasis was easily achieved by using an ultrasonic scalpel and that no major bleeding from the myocardium was observed. Similar to their report, none of our patients had massive bleeding caused by injury to the ventricular muscle or the coronary artery. Furthermore, it has been proposed that the use of an ultrasonic scalpel may help to avoid electrical stimulation of the muscles, which could otherwise lead to haemodynamic deterioration due to arrhythmia. In our experience, we did not observe any instances of ventricular arrhythmia caused by the use of an ultrasonic scalpel. Haemostasis was easily achieved by using an ultrasonic scalpel, and no major bleeding from the myocardium was observed. We did not observe major arrhythmia during the operation. No blood transfusions were required in 2 patients. Moreover, an ultrasonic scalpel can be used in patients with heavily calcified pericardium. It is very effective in dissecting the calcified pericardium from the epicardium. However, care should be taken not to apply too much pressure with an ultrasonic scalpel on the myocardium because of the potential risks of perforation.

In this study, the parameters of left ventricular diastolic function, as measured by echocardiography, exhibited a near-complete recovery to the normal range. Ghavidel *et al.* [8] suggested that left ventricular diastolic function returns to normal early after pericardiectomy in approximately 40% of patients, and late after pericardiectomy in almost 60%. In the present study, we adopted the follow-up echocardiographic data ranging from 6 to 12 months after the operation. We did this because early postoperative data are often influenced by postoperative conditions that have not sufficiently recovered.

The limitations of this clinical study are as follows: (i) the number of patients was small; (ii) we did not compare the current technique with another technique, (iii) we did not have long-term follow-up data; and (iv) we did not have data from patients undergoing other surgical methods for comparison.

## CONCLUSIONS

In conclusion, radical resection of the pericardium is essential for patients with constrictive pericarditis. The utilization of an ultrasonic scalpel is safe and effective when performing a radical pericardiectomy.

## Data Availability

The data sets presented in this article are not readily available because of local data security restrictions. Requests to access the data sets should be directed to tfukui.cvs@gmail.com
